# Behaviour of *Staphylococcus aureus* in the Rearing Substrate of *Tenebrio molitor* Larvae

**DOI:** 10.3390/vetsci10090549

**Published:** 2023-09-01

**Authors:** Francesca Pedonese, Filippo Fratini, Emma Copelotti, Francesca Marconi, Roberto Carrese, Simone Mancini

**Affiliations:** 1 Department of Veterinary Sciences, University of Pisa, Viale delle Piagge 2, 56124 Pisa, Italy; francesca.pedonese@unipi.it (F.P.); emma.copelotti@phd.unipi.it (E.C.); francesca.marconi@phd.unipi.it (F.M.); simone.mancini@unipi.it (S.M.); 2 Interdepartmental Research Center “Nutraceuticals and Food for Health”, University of Pisa, Via del Borghetto 80, 56124 Pisa, Italy

**Keywords:** mealworm, edible insect, feed, microbiological risk, *Staphylococcus aureus*

## Abstract

**Simple Summary:**

*Staphylococcus aureus* is a pathogenic microorganism of considerable importance as it is frequently involved in episodes of food poisoning due to the production of heat-resistant enterotoxins. Therefore, it becomes critically important to understand the capability of *S. aureus* to contaminate feed and food. Insects represent a new frontier in terms of feed and food production. The feed employed for rearing insects (called substrate) could represent one of the most important ways to involuntarily introduce risk in the production chain. Mealworms (*Tenebrio molitor*) represent one of the most studied insects both as feed and food. The present study assessed the ability of enterotoxigenic *S. aureus* strains to persist in the substrate and to produce enterotoxins in the reared *Tenebrio molitor* larvae. The results provide evidence of the potential risks related to the presence of this pathogen in the rearing environment.

**Abstract:**

*Tenebrio molitor* (mealworm) is one of the most promising insect species to produce sustainable feed and food with high nutritional value. Insects may harbour microorganisms both in the gut and on the exoskeleton originating from the rearing environment. *Staphylococcus aureus* is a pathogenic microorganism frequently involved in food poisoning due to its enterotoxin production. This study aimed to evaluate the *S. aureus* growth and enterotoxins production following an experimental inoculation into the *T. molitor* rearing substrate (about 7 log CFU/g). Analyses on the substrate and larvae were performed over a testing period of seven days. The microbial population dynamics were also evaluated through total viable count and lactic acid bacteria count. The effects of fasting, washing, and cooking on the microbial loads of mealworms were evaluated. The results highlighted that mealworms and substrates can maintain their microbial loads of *S. aureus* over the tested period. Moreover, fasting and washing were generally not able to significantly reduce (*p*-value > 0.05) *S. aureus* count in mealworms. On the other hand, cooking significantly reduced (*p*-value < 0.001) the microbial load in almost all cases. No production of enterotoxins was revealed during the trial. Therefore, microbiological risks can be reduced by a wise choice of substrate, appropriate control measures, and thermal treatment of larvae.

## 1. Introduction

Considering the increasingly pressing issues related to the sustainability of livestock activities in recent years, insects have received growing attention as an important source of exploitable raw materials for animal feed. Livestock sectors, such as poultry farming, pig farming, and aquaculture have implemented their studies and applications of insects as feed, mainly focusing on growth performance, microbiological and health implications, and nutrient composition [1].

Insects are a non-negligible source of protein for both humans and animals, and the percentage of edible parts is close to 100 per cent in some cases. In addition, the nutritional value of insects is much better than that of plants in terms of protein content, essential amino acids, vitamins, and minerals [2]. In the case where insects are fed to monogastric animals, growth performance and digestibility even seem to improve compared to other protein sources [3]. But the substantial aspects that most see insects as future protagonists for animal feed and human consumption are their very low environmental impact, demonstrated by negligible production of greenhouse gases, lower water use, and lower use of arable land compared to other traditionally farmed animal species [4,5,6]. What has been expressed leans toward the full sustainability of insect farming to supplement human and animal nutrition. Although their rapid development is expected soon, insects remain to date only marginally used in the feed industry, mainly because of technical, financial, and regulatory obstacles, but also because of a lack of information on the microbiological safety of the raw materials that should result from increasingly timely and detailed studies aimed at shedding light on these matters [7].

Among the many insect species, *Tenebrio molitor* (mealworm) is one of the most promising because of its high nutritional value, such as protein and fat content. Mealworms are easy to be reared with a good feed conversion ratio. Larvae of *T. molitor* could be reared on several different substrates, mostly derived from other main production activities (by-products) or waste/disposal material [8]. *T. molitor* is particularly interesting for its capacity to exploit substrates with a poor nutritional profile and a low energy intake, e.g., food leftovers and former foodstuff products [9,10]. These types of products, which often remain unused, can represent an excellent rearing substrate for insects as reported by Mancini et al. [11]. However, it is known that diet affects the nutritional characteristics of insects [12], but significant differences have also been demonstrated in the microbiological loads of insects bred on different types of substrates [13]. Mealworms showed a stable protein content regardless of diet, while modification in the fatty acids profile was revealed in relation to the substrates employed [12,14]. The use of processed animal proteins (PAPs) from seven insect species (*Hermetia illucens*, *Musca domestica*, *Tenebrio molitor*, *Alphitobius diaperinus*, *Acheta domesticus*, *Gryllodes sigillatus*, *Gryllus assimilis*) in feed for aquaculture animals was authorised in 2017 with Regulation (EU) No 2017/893 [15]. Recently, in 2021, EU Member States approved the extension of insect PAPs to poultry and swine and added an eighth species (*Bombyx mori*) with Regulation (EU) No 2021/1372 and Regulation (EU) No 2021/1925 [16,17].

To reduce the risks correlated to insect rearing, particularly microbiological risks, more relevant research studies are needed [18]. Insects can harbour microorganisms both in the gut and on the exoskeleton, although microbial contamination of insects is mainly the result of vertical transmission from mother to offspring [18,19]. In addition, insects can become contaminated by microorganisms naturally present in the substrate or rearing environment [20]. Microbiological risks can be reduced by a careful choice of the substrate and appropriate control measures in insect processing [21] especially since insects in the EU are considered farm animals and hence subjected to the EU ‘feed ban’ [22]. Fasting, washing, blanching, or cooking the insects as part of processing can reduce their viable counts and the overall microbial load [23,24,25,26].

*S. aureus* is of considerable importance as it is frequently involved in episodes of food poisoning because of the production of enterotoxins. While *S. aureus* cells are thermolabile, the staphylococcal enterotoxins are extremely heat-resistant and once present in the food cannot be inactivated by common heat treatments such as boiling, steaming, baking, or frying [27]. Therefore, it is critically important to understand how and to what extent *S. aureus* can contaminate *T. molitor* larvae and what, if any, enterotoxin production dynamics are involved in the rearing of this insect species for feed and food production.

There are a few studies available in the literature that have addressed these issues. Particularly, McGonigle et al. [28] demonstrated the ability of *S. aureus* to give rise to a persistent infection in *T. molitor*, Gorrens et al. [29] studied the growth dynamics of *S. aureus* in both rearing substrates and larvae of *Hermetia illucens,* and Cesaro et al. [18] conducted a long-term study focusing on *S. aureus* dynamics in a *T. molitor* rearing chain for food production.

The purpose of this study was to generate more data regarding the influence of *S. aureus* contamination of *T. molitor* larvae substrates on the microbial population as well as on staphylococcal enterotoxin production in the reared larvae.

## 2. Materials and Methods

### 2.1. Experimental Design

**Figure 1:**
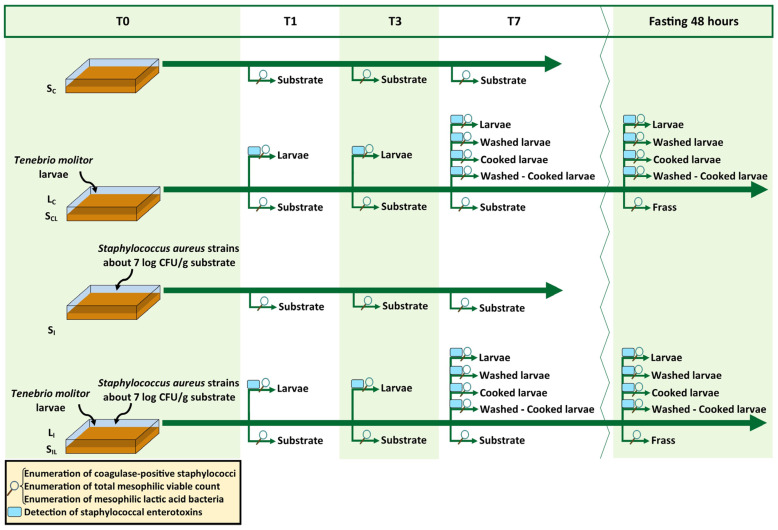
Experimental design of the trial. Starting time T0; Sampling time after 1, 3, and 7 days (T1, T3, and T7); fasting 48 h: sampling of fasted larvae derived from T7; S_C_: control substrate; L_C_: control larvae; S_CL_: control substrate with larvae; S_I_: inoculated substrate; L_I_: inoculated larvae; S_IL_: inoculated substrate with larvae.

*Tenebrio molitor* larvae were reared at the Department of Veterinary Sciences of the University of Pisa, in plastic crates of 39 × 28 × 14 cm. The farming temperature was set to 25 °C (±1) with a relative humidity range of 55–65%. The larvae were fed a mix of 1:1 spent brewery grains and bread leftover (dry matter, DM: 97.08%; ether extract: 2.26% of DM; crude protein: 14.20% of DM; ash: 2.47% of DM). The last instar larvae were sieved and fasted for two days before starting the trial. Larvae and substrates were previously analysed to determine, as described in Section 2.3, the initial count of coagulase-positive staphylococci (CoPS), that resulted under the detection limit, and to check the load of total mesophilic viable count (TVC) and total mesophilic lactic acid bacteria (LAB) before the beginning of the trial (T0). Larvae were also tested for staphylococcal enterotoxins at T0 (see Section 2.4) giving negative results. A detailed description of the experimental design can be found in Figure 1. Four different sets of crates (in triplicate) were employed as only control substrate (S_C_, spent brewery grains: bread leftovers, 1:1), only *S. aureus* inoculated substrate (S_I_), control substrate with larvae (named as S_CL_ for the substrate, and L_C_ for the larvae), and *S. aureus* inoculated substrate with larvae (named S_IL_ for the substrate and L_I_ for the larvae). The ratio of substrate–larvae was 5:1. Where needed, (S_I_ and S_IL_) the rearing substrate was experimentally inoculated with an *inoculum* composed of two strains of *S. aureus* (see section below). Samples of larvae and substrates were collected after 1, 3, and 7 days (T1, T3, and T7) to determine the microbiological loads of CoPS, TVC, and LAB, and limited to larvae samples, the production of staphylococcal enterotoxins. Furthermore, the effects of fasting, washing, and cooking were tested (on day 7) performing the same analyses described above. Fasting was conducted for 48 h in sterile plastic containers with a net as the base to avoid faecal contact and the frass was collected in a second sterile plastic container placed below. Washing was performed in sterile bags with sterile saline solution (9:1, *v*/*w*). Bags were manually shaken for 3 min, then the washing solution was removed by pipetting and collected. Lastly, larvae were cooked in a preheated oven at 150 °C for 10 min in aluminium trays.

### 2.2. Bacterial Strains and Preparation of the Experimental Inoculum

Two reference methicillin-susceptible *Staphylococcus aureus* strains were used for the experimental inoculation tests: *S. aureus* NCTC 10652 (reference strain for staphylococcal enterotoxin A and D production) and *S. aureus* ATCC 25923, harbouring *seg* and *sei* genes [30] and slight enterotoxin producers in preliminary tests (see Section 2.4). They were stored at −80 °C in Brain Heart Infusion Broth (Oxoid, Basingstoke, UK), supplemented with 15% glycerol as a cryoprotectant, and were revitalised in the same medium for 24 h at 37 °C under aerobic conditions. The two strains were prepared separately to obtain the final *inoculum*. Each bacterial broth culture was centrifuged at 6000 rpm for 10 min and the supernatant was discarded. Subsequently, three washing steps with a sterile saline solution followed by centrifugation, as above described, were performed. Then, the individual pellets were collected in 10 mL of sterile saline solution, and the suspensions of the two strains were combined in equal amounts to obtain the final mix used for inoculation (about 8–9 log CFU/mL). Based on preliminary tests, the final amount of *inoculum* was determined to obtain a staphylococcal count between 6 and 7 log CFU per g of substrate.

### 2.3. Microbiological Analyses

Sampled substrates and larvae were weighed into sterile Stomacher bags (Seward Ltd., Worthing, UK), and diluted with sterile saline solution (9:1, *v*/*w*). The prepared samples were thoroughly cracked and homogenised for 60 s in a Stomacher 400 Circulator Lab Blender (Seward Ltd.). Serial 10-fold dilutions in saline solution were applied and the following enumerations were carried out following the relevant ISO: CoPS count on Baird Parker Agar (ISO 6888-1:1999/A2:2018) [31], TVC (ISO 4833-1:2013) [32], LAB count (ISO 15,214:1998) [33]. Counts below the detection limit were indicated as 1.00 log CFU/g for CoPS and 0.50 log CFU/g for TVC and LAB.

### 2.4. Detection of Staphylococcal Enterotoxins

Staphylococcal enterotoxin determinations were performed on the two *S. aureus* strains, on the *inoculum*, and on the larvae samples (from T1 to T7, considering the steps of fasting, washing, and cooking). A commercial enzyme immunoassay for the combined analysis of staphylococcal enterotoxins (A-E) in food (RIDASCREEN Set Total, r-Biopharm, Darmstadt, Germany) was used. Larvae samples were stored at −20 °C and were thawed at refrigeration temperature before use. For the *inoculum*, the *S. aureus* strains were revitalised as described above. The broth culture was then centrifuged at 3500 rpm for 5 min and the supernatant was filtered through bacteriological syringe filters with a pore size of 0.20 μm. Then, the test was performed following the manufacturer’s instructions. The absorbance was measured at 450/620 nm with a microplate reader (Multiskan FC Microplate Photometer, Thermo Fisher Scientific, Ratastie, Finland). The preliminary detection on the *S. aureus* NCTC 10652 and *S. aureus* ATCC 25923, as well as on the mix (*inoculum*), showed the production of staphylococcal enterotoxins and evidenced that the first strain was a strong producer of enterotoxins while the second strain was a mild one. As a mix of the strains, the *inoculum* exhibited a high production of enterotoxins.

### 2.5. Statistical Analysis

R free statistical software was used [34]. The time effect was tested via one-way ANOVA on the determination of CoPS, TVC, and LAB in relation to the *inoculum* and the presence of the larvae (S_C_, S_CL_, S_I_, and S_IL_). The effect of the presence of the larvae, on the microbial growth, was tested via the Student T test (S_C_ vs. S_CL_; S_I_ vs. S_IL_). Similarly, the effect of the *inoculum* was tested via the Student T test (S_C_ vs. S_I_; S_CL_ vs. S_IL_; L_C_ vs. L_I_). The effects of fasting, washing, and cooking were tested only on the larvae microbial counts in relation to the *inoculum* via the Student T test. Statistical significance was set at 0.05 and differences were assessed using Tukey’s test.

## 3. Results

### 3.1. Staphylococcus aureus Growth

The results at T0 highlighted that CoPS were under the detection limit in S_C_, L_C_, and S_CL_. During the seven days of the trial, CoPS were constantly below the detection limit in S_C_, S_CL_, and L_C_. The inoculated substrate (S_I_) showed a significant decrease in the CoPS count from T0 to T7 with mixed values at T1 and T3 (see Table 1). A similar trend, even if not significant, was detected in the inoculated substrate used as feed for the larvae (S_IL_), where CoPS decreased over time. The larvae fed the inoculated substrate (L_I_) were shown to be affected by the *inoculum* reaching atT3 the highest value of CoPS that was maintained without significant changes until T7, even if a decreasing trend was shown (Table 1). Regarding the presence of the larvae, the lack of CoPS in both the control substrate and the T0 larvae affected the detection of these bacteria in S_C_ and S_CL_, while the presence of the *inoculum* in S_I_ and S_IL_ showed that the larvae did not affect the counts of CoPS at T1 and T3. At T7 the presence of the larvae in the S_IL_ decreased the count of the CoPS resulting in a statistical difference from S_I_ (Table 1, *p*-value 0.033). As expected, the effect of the *inoculum* was significant in all the relevant comparisons as presented in Table 1. No mortality during the whole trial was recorded.

### 3.2. Total Mesophilic Viable Count Growth

At the beginning of the trial (T0) TVC of the not inoculated substrate (S_C_ and S_CL_) was 3.44 log CFU/g, while the larvae (L_C_ and L_I_) showed a TVC of 7.48 log CFU/g. The addition of the inoculum at T0 in the substrate caused an increase in the TVC in S_I_ and S_IL_ (see Table 2). During the trial, no significant variation of TVC was recorded in S_C_, S_I_, L_C_, and L_I_. The control substrate used for rearing the larvae (S_CL_) showed a significant increase (Table 2, *p*-value < 0.001) of TVC at T1, without a further significant increase at T3 and T7. The TVC in S_IL_ showed a significant increase only at T7. Regarding the effect of the larvae, the presence of mealworms in the control substrate increased the TVC loads at all the tested times. In the inoculated crates, the effect of the presence of the larvae was significant only at T7 (Table 2). As reported above for the CoPS, an expected increase of TVC was reported between S_C_ and S_I_ concerning the addition of the *inoculum* in the substrate. The addition of the *inoculum*, in the crates with the larvae, affected the substrates’ TVC at T3 and T7. No changes in TVC were detected at T1 between S_CL_ and S_IL_. Similarly, no differences were reported for TVC in the larvae samples (L_C_ and L_I_) during the whole trial.

### 3.3. Mesophilic Lactic acid Bacteria Growth

Determination of LAB in the substrate (S_C_, S_CL_, S_I_, and S_IL_) at T0 showed a load of 0.83 log CFU/g. The larvae (L_C_ and L_I_) showed a LAB content of 6.80 log CFU/g. No significant changes were detected in LAB loads in S_C_ and L_I_ during the whole experiment (Table 3), while significant increases of LAB were highlighted at T1 in the other substrates with different magnitudes concerning the presence of the larvae or the *inoculum* (S_CL_, S_I_, and S_IL_; Table 3). Larvae fed by the control substrate showed a non-linear effect of time during the trial. Indeed, the LAB loads decreased between T0 and T3, while an increase was detected at T7 reaching the same amount of LAB evaluated at T0. Regarding the larvae’ LAB load, the presence of mealworms in the crates significantly increased the LAB contents in the substrate (Table 3). A positive effect of the *inoculum* was detected in the LAB counts of the inoculated substrate without larvae (S_I_), resulting in a significant difference between T0 and the other times. No variations in relation to the *inoculum* were revealed among the other samples (S_CL_, S_IL_, L_C_, and L_I_).

### 3.4. Effect of Fasting, Washing, and Cooking on Microbiological Counts

It was observed that fasting for 48 h had a low effect on the counts of CoPS, TVC, and LAB of the larvae, that were both fed the control and the inoculated substrates (Table 4). Indeed, only the control-fed larvae were shown to be significantly affected by fasting in relation to the LAB counts. The analyses carried out on the frass showed that the *inoculum* affected the CoPS count in the larvae faeces (4.91 log CFU/g in the frass of L_I_, while 1.00 log CFU/g in the frass of L_C_), small changes were also highlighted in LAB counts (8.05 log CFU/g and 7.73 log CFU/g respectively for L_C_ and L_I_); no variation was recorded in frass TVC in relation to the *inoculum* (9.16 log CFU/g and 9.01 log CFU/g respectively for L_C_ and L_I_).

The results of the washing treatment showed no statistical effect on the larvae microbiological counts (both fed controlled and inoculated substrates). Indeed, the variation in microbial counts was restricted to the range of 0.50 log CFU/g; while the washing solution (sterile saline solution used for the washing step) showed, as average, CoPS count of 1.28 log CFU/g, TVC of 4.90 log CFU/g and LAB count of 3.85 log CFU/g. Cooking larvae for 10 min at 150 °C mostly reduced CoPS, TVC, and LAB with statistically significant reductions (Table 5). Only L_I_ samples after fasting and washing showed to be not significantly affected by cooking in relation to CoPS determination.

### 3.5. Enterotoxins Production

All the larvae samples, without relation to the *inoculum*, the time, the fasting, the washing, the cooking, or their interactions, showed no enterotoxin production.

## 4. Discussion

The microbiota of insects that can be used as feed and food is complex and only partially explored [35]. The presence and growth capacity of pathogenic microorganisms deriving directly from insects, rearing substrate, or the processing cycle must be considered along the whole production chain. Among these microorganisms, spore-forming ones such as *Bacillus cereus* and pathogenic *Clostridium* spp. are of major importance, as well as *S. aureus*, which is non-spore-forming but able to produce enterotoxins with high stability to post-harvest treatments [36].

The present study assessed the ability of the used enterotoxigenic *S. aureus* strains to grow in larvae and substrates and to produce enterotoxins in the tested *T. molitor* larvae. As reviewed by Garofalo et al. [19], the genus *Staphylococcus* is commonly found in edible insects with many different species, among which *S. aureus*, which can also be found in insects rearing substrates [29]. According to Kooh et al. [21], it is necessary to take some control measures to prevent *S. aureus* contamination and growth, which in edible insects may be caused by handling or processing. Furthermore, the Guide on Good Hygiene Practices, redacted by IPIFF (International Platform of Insects for Food and Feed) [37], highlights that *S. aureus* is considered one of the hazards to be kept monitored as an indicator of hygiene, raw materials manipulation, and processing operations (limit 2 log CFU/g). In this study, we did not apply any resuscitation step or other methods to detect viable but nonculturable bacteria (VBNC); it is well known that VBNC pathogenic bacteria, including *S. aureus*, are considered a threat to public health and food safety and they have been implicated as the causative agent in several food disease outbreaks [38]. This aspect deserves further study.

Based on preliminary tests, we used an experimental *S. aureus inoculum* of 6–7 log CFU/g to contaminate the substrate corresponding to a contamination of about 2 log CFU/g lower in the larvae. This value of *inoculum* is suitable to appreciate the *S. aureus* count increases up to the level correlated with enterotoxin production (10^5^ to 10^6^ CFU per g, as reported by Bennett et al. [39]). Cesaro et al. [18] in a similar study used the same *inoculum* and obtained *S. aureus* counts on larvae samples under the detection limit within a time of 70 days. Nevertheless, the two studies were not exactly comparable, as Cesaro et al. [18] studied the whole life cycle of *T. molitor* starting from the eggs, and sampled larvae not before day 28. In contrast, we performed analyses on the last larvae instar, which are usually employed for food and feed. Our study provides results concerning the behaviour of *S. aureus* in larvae and substrates in the first 7 days after inoculation. It shows that larvae can become rapidly contaminated in the inoculated substrate and can maintain the microbial loads over the test period. Even fasting and washing were not able to significantly reduce the *S. aureus* amount in *T. molitor* larvae.

These data appear to be in line with those reported by McGonigle et al. [28] who recovered *S. aureus* viable cells in intracellular and extracellular fractions of *T. molitor* for 21 days after an experimental inoculation. The decreasing *S. aureus* trend from T1 to T7 in inoculated substrates agrees with Gorrens et al. [29], who reported that even if in a different rearing substrate and a different insect species (*Hermetia illucens*), an average *S. aureus* count ≤6.2 log CFU/g at day 6 with an initial *inoculum* of about 7 log CFU/g of the substrate. Furthermore, Cesaro et al. [18] data testified a decrease from T0 to T14 (with a count of about 6 log CFU/g) starting from the same *inoculum* in inoculated rearing substrates. Gorrens et al. [29], similarly to Cesaro et al. [18], found *S. aureus* counts in larvae constantly under the detection limit; instead, our results show a constant *S. aureus* load during all the trials. Considering the variety of insects and substrates that can be used as feed and food and all the possible interactions with microorganisms, the behaviour of different strains of *S. aureus* in the larvae deserves further investigation. Our data on staphylococcal enterotoxin production in larvae reared on an inoculated substrate confirm those of Cesaro et al. [18] and are in line with the results of the *S. aureus* count, that was under 5 log CFU/g. The analytical determination was not performed on the substrate samples. However, the absence of enterotoxins in the substrate could be hypothesised as the larvae samples showed negative results and *S. aureus* counts of the substrate were stable during the tested time, without any growth.

Larvae microbiota influenced the TVC loads of the control substrate. Similar values were reported by Mancini et al. [11], who obtained a TVC of 7.08 and 7.63 log CFU/g in larvae reared in brewery-spent grain and bread, respectively. TVC values were also influenced by the presence of the *inoculum*. Concerning the quantification of LAB loads, our results show an incrementing trend in the substrate when larvae were present. Analysing mealworm microbiota, Stoops et al. [40] highlighted that LAB represents an abundant community and similar results were reported by Osimani et al. [20] and Vandeweyer et al. [41]. It is important to highlight that LAB could also act as a probiotic and produce natural antimicrobial compounds such as bacteriocins, organic acids, and small molecules (e.g., H_2_O_2_, diacetyl) active against a wide range of microorganisms [42]. Some evidence of this capability was reported by Lecocq et al. [43] who added to the diet of mealworms a *Pediococcus pentosaceus* strain isolated from the gut of the larvae. Indeed, Lecocq et al. [43] reported a significant beneficial effect on larval growth rate and survival into adulthood.

In our trial, no mortality of larvae was observed throughout the rearing period, thus suggesting that the inoculation of *S. aureus*, at least with the employed strains, in the rearing substrate did not reduce the viability of mealworms, despite the initial contamination level, as previously found by Cesaro et al. [18].

Fasting was performed to empty the intestinal content of insects and limit microbial counts [44], while washing was used to reduce the external microbial contamination of larvae [45]. Our results showed a not significant impact of these treatments on *S. aureus* loads, as demonstrated previously for *Listeria monocytogenes* [23,45,46].

Interestingly, a CoPS count of about 5 log CFU/g was detected in the frass of larvae reared in inoculated substrates, as also previously reported by Cesaro et al. [18]. It attests that frass can be a potential carrier of this microorganism, and therefore it must be appropriately treated before use as reported in Regulation (EU) No 2021/1925 [17].

Furthermore, for TVC, fasting and washing results did not show a significant effect of the treatments, as reported by Wynants et al. [23]. Conversely, Pöllinger-Zierler et al. [44] evidenced a significant effect of starvation for 24 h with a reduction of the mealworm larvae TVC of about 1 log CFU/g. A decrease in LAB count was detected after fasting in relation to a high count of these bacteria in the frass. As reported for CoPS, LAB and TVC counts in frass were highly represented (7.89 and 9.08 log CFU/g for LAB and TVC, respectively) confirming that frass can be a potential source of several different microbial communities.

Even if cooking could determine changes in the insects’ nutritional profile [12], it represents the best treatment to reduce microbial load and the connected risk to human or animal health, as for other products of animal origin. Our results confirmed the effectiveness of the oven cooking treatment with a combination time-temperature of 10 min at 150 °C. In detail, the CoPS counts were reduced under the detection limit, while the decreases ranged between 4.45–6.13 and 4.55–6.61 log CFU/g for TVC and LAB counts, respectively. Likewise, using the same cooking treatment, Mancini et al. [25] reported a drastic reduction of *L. monocytogenes* in *T. molitor* larvae experimentally fed with an inoculated substrate. A similar TVC decrease (4.74 log CFU/g) was reported by Pöllinger-Zierler et al. [44] by cooking mealworms in a microwave at 850 W for 10 min. Compared to Caparros Megido et al. [26] findings, our results showed a higher decrease in TVC, mainly because of the cooking parameters, as they used a low-temperature treatment (70 °C) for 15 and 30 min leading to a maximum TVC reduction of 2.4 log CFU/g.

## 5. Conclusions

These results highlighted that *S. aureus* could persist in the rearing substrates of mealworm larvae and be a potential risk for the production of feed and food. Heat treatments could be fundamental steps during the production cycle, mostly in relation to the final product characteristics, and as a safety procedure to lower the risk associated with human and animal health. Specifically concerning *S. aureus* contamination, other control measures can consist of reinforcing biosecurity and hygiene measures for personnel, as well as applying adequate cleaning and disinfection protocols at various levels of the feed or food production chain.

## Figures and Tables

**Table 1:** Effects of time, larvae, and *inoculum* on the coagulase-positive staphylococci count (log CFU/g).

*Effect of Time (Days)*	T0	T1	T3	T7	RMSE	*p*-Value
S_C_	1.00	1.00	1.00	1.00	-	-
S_I_	6.92 ^a^	6.44 ^ab^	6.55 ^ab^	6.21 ^b^	0.221	0.026
S_CL_	1.00	1.00	1.00	1.00	-	-
S_IL_	6.92	6.28	6.42	6.09	0.319	0.064
L_C_	1.00	1.00	1.00	1.00	-	-
L_I_	1.00 ^b^	4.18 ^a^	4.54 ^a^	3.62 ^a^	0.495	<0.001
***Effect of larvae (p-value)***						
S_C_ vs. S_CL_		-	-	-		
S_I_ vs. S_IL_		0.346	0.641	0.033		
***Effect of inoculum (p-value)***						
S_C_ vs. S_I_		<0.001	<0.001	<0.001		
S_CL_ vs. S_IL_		<0.001	<0.001	<0.001		
L_C_ vs. L_I_		<0.001	<0.001	<0.001		

L_C_: control larvae; L_I_: inoculated larvae; S_C_: control substrate; S_CL_: control substrate with larvae; S_I_: inoculated substrate; S_IL_: inoculated substrate with larvae. RMSE: root mean square error. ^a^, ^b^ in the same row indicates significant differences at *p* < 0.05.

**Table 2:** Effects of time, larvae, and *inoculum* on the Total Mesophilic Viable count (log CFU/g).

*Effect of Time (Days)*	T0	T1	T3	T7	RMSE	*p*-Value
S_C_	3.44	3.44	3.44	3.44	-	-
S_I_	7.18	6.58	6.79	6.50	0.328	0.124
S_CL_	3.44 ^b^	6.25 ^a^	6.60 ^a^	6.84 ^a^	0.480	<0.001
S_IL_	7.18 ^b^	6.58 ^b^	7.10 ^b^	8.13 ^a^	0.412	<0.001
L_C_	7.48	7.44	7.43	7.84	0.331	0.278
L_I_	7.48	7.58	7.48	8.07	0.360	0.109
***Effect of larvae (p-value)***						
S_C_ vs. S_CL_		0.004	0.003	0.003		
S_I_ vs. S_IL_		0.962	0.130	0.005		
***Effect of inoculum (p-value)***						
S_C_ vs. S_I_		0.002	0.002	0.004		
S_CL_ vs. S_IL_		0.133	0.014	0.011		
L_C_ vs. L_I_		0.642	0.895	0.102		

L_C_: control larvae; L_I_: inoculated larvae; S_C_: control substrate; S_CL_: control substrate with larvae; S_I_: inoculated substrate; S_IL_: inoculated substrate with larvae. RMSE: root mean square error. ^a, b^ in the same row indicates significant differences at *p* < 0.05.

**Table 3:** Effects of time, larvae and *inoculum* on the mesophilic lactic acid bacteria counts (log CFU/g).

*Effect of Time (Days)*	T0	T1	T3	T7	RMSE	*p*-Value
S_C_	0.83	0.83	0.83	0.83	-	-
S_I_	0.50 ^b^	1.53 ^a^	1.49 ^a^	2.27 ^a^	0.321	0.001
S_CL_	0.83 ^b^	4.68 ^a^	5.42 ^a^	5.23 ^a^	0.654	<0.001
S_IL_	0.83 ^b^	4.42 ^a^	4.74 ^a^	5.49 ^a^	0.545	<0.001
L_C_	6.80 ^a^	6.64 ^ab^	5.88 ^b^	6.80 ^a^	0.393	0.040
L_I_	6.80	6.83	6.67	7.03	0.295	0.425
***Effect of larvae (p-value)***						
S_C_ vs. S_CL_		<0.001	0.002	0.001		
S_I_ vs. S_IL_		0.002	0.001	0.004		
***Effect of inoculum (p-value)***						
S_C_ vs. S_I_		0.071	0.092	0.003		
S_CL_ vs. S_IL_		0.438	0.337	0.710		
L_C_ vs. L_I_		0.414	0.071	0.218		

L_C_: control larvae; L_I_: inoculated larvae; S_C_: control substrate; S_CL_: control substrate with larvae; S_I_: inoculated substrate; S_IL_: inoculated substrate with larvae. RMSE: root mean square error. ^a, b^ in the same row indicates significant differences at *p* < 0.05.

**Table 4:** Effect of fasting on controlled and inoculated larvae bacterial counts (log CFU/g).

		*Unfasted*	*Fasted*	RMSE	*p*-Value
L_C_	CoPS	1.00	1.00	-	-
	TVC	7.84	7.72	0.168	0.352
	LAB	6.80	6.11	0.233	0.006
L_I_	CoPS	3.59	3.20	0.522	0.349
	TVC	8.07	7.89	0.167	0.196
	LAB	7.03	7.07	0.218	0.814

L_C_: controlled larvae; L_I_: inoculated larvae. CoPS: coagulase-positive staphylococci; TVC: total mesophilic viable count; LAB: mesophilic lactic acid bacteria. RMSE: root mean square error.

**Table 5:** Effect of cooking on controlled and inoculated larvae bacterial counts (log CFU/g).

*Fasting*	*Washing*	*Inoculum*	*Bacteria*	*Raw*	*Cooked*	RMSE	*p*-Value
Unfasted	Unwashed	L_C_	CoPS	1.00	1.00	-	-
			TVC	7.84	3.04	0.339	<0.001
			LAB	6.80	1.48	0.356	<0.001
	Washed		CoPS	1.00	1.00	-	-
			TVC	8.08	1.95	0.609	<0.001
			LAB	7.11	0.93	0.367	<0.001
	Unwashed	L_I_	CoPS	3.59	1.00	0.482	<0.001
			TVC	8.07	2.68	0.519	<0.001
			LAB	7.03	1.67	0.430	<0.001
	Washed		CoPS	3.15	1.00	0.439	0.004
			TVC	7.77	1.67	0.419	<0.001
			LAB	6.96	0.99	0.354	<0.001
Fasted	Unwashed	L_C_	CoPS	1.00	1.00	-	-
			TVC	7.72	3.27	0.694	0.001
			LAB	6.11	1.56	0.389	<0.001
	Washed		CoPS	1.00	1.00	-	-
			TVC	7.85	2.12	0.401	<0.001
			LAB	7.11	0.50	0.333	<0.001
	Unwashed	L_I_	CoPS	3.20	1.00	0.246	<0.001
			TVC	7.89	2.78	0.043	<0.001
			LAB	7.07	1.03	0.398	<0.001
	Washed		CoPS	1.62	1.00	0.753	0.374
			TVC	7.50	2.07	0.443	<0.001
			LAB	6.55	0.67	0.282	<0.001

L_C_: control larvae; L_I_: inoculated larvae. CoPS: coagulase-positive staphylococci; TVC: total mesophilic viable count; LAB: mesophilic lactic acid bacteria. RMSE: root mean square error.

## Data Availability

The data presented in this study are available on request from the corresponding author.
